# Cosmetic Products with Potential Photoprotective Effects Based on Natural Compounds Extracted from Waste of the Winemaking Industry

**DOI:** 10.3390/molecules29122775

**Published:** 2024-06-11

**Authors:** Ana-Maria Draghici-Popa, Diana-Ioana Buliga, Ioana Popa, Stefan Theodor Tomas, Raluca Stan, Aurelian Cristian Boscornea

**Affiliations:** Faculty of Chemical Engineering and Biotechnologies, National University of Science and Technology Politehnica Bucharest, 1–7 Gheorghe Polizu St., 1st District, 011061 Bucharest, Romania; ana_maria.draghici@upb.ro (A.-M.D.-P.); ioana.asofiei@upb.ro (I.P.); stefan.tomas@upb.ro (S.T.T.); raluca.stan@upb.ro (R.S.)

**Keywords:** sunscreen, grape marc, polyphenols, antioxidants, photoprotection, sunburn, UV rays

## Abstract

Grape marc is a by-product resulting from the winemaking industry that still contains beneficial compounds that can be valorized. Thus, we report here the possibility of using polyphenolic extracts of grape marc origin to obtain sun protection creams. The extractions were performed in ethanol and acetone solutions using pomace from different grape varieties (Merlot, Bläufrankisch, Fetească Neagră, Isabella) as a raw material. The obtained extracts were analyzed in order to determine the total phenolic content, the antioxidant activity, and the sun protection factor (SPF) via Mansur spectrophotometric assay. The best results were achieved using 70% ethanol in water as a solvent. The extracts with the highest potential photoprotective effects are from the Merlot variety (SPF_spectrophotometric_ = 7.83 ± 0.76). The sunscreens were prepared using the 70% ethanolic extract of the Merlot variety evaporated to dryness, redissolved in either distilled water or ethanol. The SPF estimated in vitro via the COLIPA method showed values of 14.07 ± 1.50 and 11.46 ± 1.32 for the aqueous and ethanolic extracts, respectively, when working with a cream to polyphenolic extract a ratio of 1/1 (*w*/*w*). At the same time, the use of aqueous polyphenolic extracts ensures the better stability of creams compared with the ethanolic ones.

## 1. Introduction

Solar energy represents the driving force for the development of life on Earth. The sun produces an enormous amount of energy including cosmic energy, gamma rays, X-rays, ultraviolet (UV) radiation, visible radiation, and infrared radiation [1,2,3]. As emitted from the sun, light contains three types of UV radiation: UVC (100–290 nm), UVB (290–320 nm), and UVA (320–400 nm). However, the ozone layer absorbs 100% of UVC, 90% of UVB, and a negligible amount of UVA [2]. Several epidemiological studies have provided evidence for the impacts of the beneficial and harmful effects of sunlight, especially solar UV radiation exposure, on overall human health status [4]. The human skin is continuously exposed to UV radiation, leading to damaging effects that can be categorized into acute damages, such as sunburn, erythema, pain oedema and photodermatoses, and more serious chronic damages, including photoageing and premalignant skin lesions, such as actinic keratosis and skin cancers, i.e., basal cell carcinoma, squamous cell carcinoma, and malignant melanoma [5]. UVA rays penetrate the deeper layers of skin (the dermis) and interact with skin cells and generate free radicals. The latter are highly reactive oxygen molecules and also known as ROS—reactive oxygen species. The body uses antioxidants to neutralize these potentially harmful free radicals. When there is an imbalance between the production of free radicals and the body’s ability to neutralize them, the free radicals start to damage cells in a process known as oxidative stress [6]. UVA rays are most commonly associated with photoaging (premature skin aging caused by the sun), sun allergies, and hyperpigmentation, such as sun spots (also known as age spots) [7,8,9,10]. 

UVB rays provide the energy needed by the skin to produce Vitamin D and stimulate the production of melanin, which is responsible for tanning [11]. They only penetrate the outermost layers of skin (the epidermis), but they cause more immediate damage, such as sunburn and temporarily thickened skin [12]. Sunburn is described as the erythematic acute cutaneous reaction in response to increased melanin and the apoptosis of keratinocytes to prevent skin carcinoma [13]. Sunburns are conditions caused by excessive exposure to UV radiation from sunlight or artificial sources, such as tanning beds and booths [14,15]. UVB rays are directly absorbed by cellular DNA and can lead to skin diseases such as actinic keratosis and skin cancer [16]. UVB causes sunburns—the biggest risk factors being exposure time and UV rays intensity—and DNA strand breakage [17]. It is also responsible for pyrimidine dimer changes that are associated with non-melanoma skin malignancies [18,19]. 

As solar radiation is a major risk factor for malignant melanoma, it can be concluded that lowering sun exposure through the topical use of sunscreens could be associated with reduced disease risk [1,17]. Photoprotection recommendations include the use of photoprotective outerwear; behavioral modifications, such as shade seeking; and daily use of broad-spectrum sunscreen [20]. A sunscreen is a compound (or lotion) that uses agents to block, deflect, or reflect the sun’s harmful UV rays. UV filters, the active ingredients in sunscreens, are classified as organic (“chemical”) or inorganic (“physical” or “mineral”) based on their mechanism of action [21].

Photoprotection creams include both essential and secondary protection options. The primary alternatives are sunscreens that contain inorganic filters that reflect and dissipate light or organic filters that absorb UV radiation [22]. Secondary components contain cancer-preventing agents, osmolytes, and DNA-repair enzymes which help to limit skin damage by inhibiting photochemical processes caused by UV radiation [19,23,24,25].

Titanium dioxide and zinc oxide, as inorganic filters, have been considered highly protective agents, and their combination is particularly valuable due to its ability to filter both UVA and UVB radiations, providing a greater protection range compared with the individual components [26]. Compounds used as inorganic filters are often processed into nanoscale particles to improve the texture, coverage, and feel of sunscreen on the skin while maintaining the optical properties needed to protect the skin from UV. There is a perceived risk of these nanoparticles penetrating the viable layers of the skin [27], despite evidence to the contrary [28]. Inorganic filters in nanometric form are also used in sunscreens as substitutes for synthetic organic filters in products defined as green or ecological [29]. Recent studies on ZnO and TiO_2_ nanoparticles have reported multiple adverse effects on a wide range of marine microorganisms [30,31], while these effects are not observed for non-nano inorganic filters [32].

Organic filters are soluble aromatic compounds that contain in their structure a system of conjugated π electrons. They absorb UV radiation and are promoted from the fundamental state to an excited molecular state. When returning to the fundamental state, the absorbed energy is dissipated as heat [33,34,35]. There is also the possibility of converting the absorbed energy into radiation of greater wavelength but lower energy [36,37,38,39].

UV absorbing agents must accumulate in the upper layers of the skin to provide a dense coating of light absorption and guarantee water resistance [35]. Organic sunscreens are usually alcohol-based, lipophilic, or a combination of both, which can facilitate the delivery of active substances into the stratum corneum. However, there is a risk that the long residence times of the active substances in the skin may lead to unwanted penetration into the living skin, which could adversely affect the biology of the skin. Studies conducted on approved organic filters indicate that at an application rate of approximately 2 mg/cm^2^, the penetration is minimal [40].

Currently, to optimize the sun protection and photostability of sunscreens, natural antioxidants are used [41,42,43,44]. Scientific evidence has shown the benefits of topical and oral use of polyphenols from certain plant species against UV radiation [45]. Polyphenols are antioxidant molecules that, similar to vitamins and antioxidant enzymes, help with preventing oxidative stress caused by reactive oxygen species. The antioxidant properties of polyphenols are primarily due to the presence of hydroxyl groups [46]. In addition to their antioxidant properties, polyphenols can act as enzyme inhibitors or inducers, impacting anti-inflammatory pathways [47]. Incorporating antioxidants into photoprotection cosmetics could provide additional benefits by scavenging free radicals. Areas of skin that were pretreated with natural polyphenolic extracts prior to UV exposure developed less erythema and were found to have less sunburn upon microscopic examination [48]. Natural polyphenolic extracts have also been found to reduce the damaging effects of UV radiation on Langerhans cells, a subpopulation of epidermal cells known to play a key role in the development of skin-cell-mediated immune responses [49]. The extracts were effective at reducing the erythema response to both UVB and UVA radiation. Polyphenols are a category of compounds naturally found in plant foods, such as fruits, vegetables, herbs, spices, tea, dark chocolate, and wine. Grape marc is a rich source of polyphenols [50,51,52,53,54]. This is a by-product resulting from grape processing in the winemaking industry. It is a solid heterogeneous mixture, consisting mainly of skins (63%), seeds (33%), and scraps from the pulp [55].

**Figure 1:**
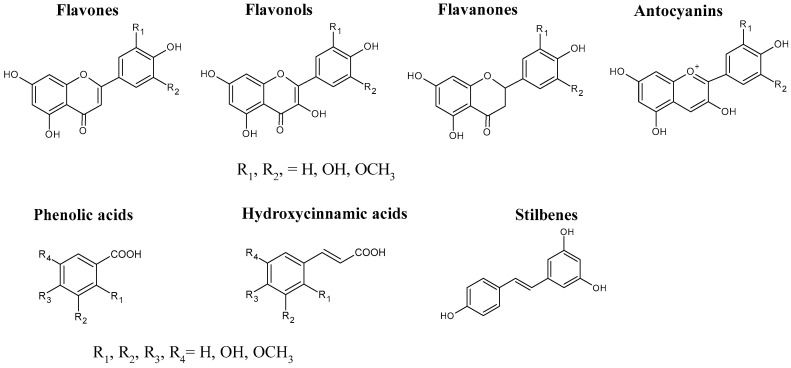
Phenolic compounds present in grapes.

Depending on the type of crusher or press used to extract the must, pomace represents between 20 and 40% of the grapes’ weight. It is estimated that for grape production of 7–14 t/ha, approximately 2–4 t/ha of pomace are generated (roughly 4.5–9.0 m^3^), with an organic matter content of 0.9–1.8 t/ha [56]. The phenolic composition of grape marc varies greatly depending on the grape variety, environmental and climate conditions, soil type, degree of ripeness, and winemaking process. Phenols have a common structure comprising an aromatic benzene ring with one or more hydroxyl substituents. Flavonoids have three cycles in their structure and are the most abundant phenolic compounds found in both grapes and wines [57]. This family of molecules consist of different sub-categories such as flavones, flavonols, flavonones, and anthocyanins (Figure 1). To these molecules, the non-flavonoid phenolic compounds, including phenolic acids (hydroxybenzoic acids), hydroxycinnamic acids, and stilbenes, are added [58]. 

The specialized literature attributes the antioxidant activity of grapes to the fruit’s skin, with 90% of it due to the presence of anthocyanins and proanthocyanins and 10% due to flavonols, flavanols, and phenolic acids [59]. In vivo and in vitro studies performed with grape seeds assign the antioxidant capacity to flavonoids [60].

This study presents alternatives for the extraction of polyphenolic compounds from grape marc of several varieties. Their further characterization and testing to obtain cosmetic products with a potential photoprotective effect was performed. The characterization of the polyphenolic extracts was carried out by determining the OD280 index, the total phenolic content, and the antioxidant activity via the CUPRAC assay. The analysis of the photoprotective effect was carried out in vitro by Mansur [61] and using the COLIPA method (European Cosmetic, Toiletry, and Perfumery Association) [62,63]. The importance and novelty of the study lie in the partial recovery of waste generated by the wine industry, which is available in large quantities, for a modern area with a great commercial impact: cosmetic products. Although the use of polyphenols from various sources in the production of cosmetics is a widely addressed field at the moment, the production of lotions with photoprotective effects is less investigated. This study also aimed to find extraction options for compounds that have good-to-excellent absorption in the UVB and UVA ranges, considering that most polyphenols primarily absorb at wavelengths below 300 nm. The method of investigating the photoprotective effect aimed to find simple, efficient, and inexpensive methods that allow the rapid characterization of the obtained extracts for use in sun protection products. Considering the diverse composition of pomace and the multitude of factors that influence it, the results of this study provide the opportunity for a quick analysis and decision regarding the valorization of this waste from the winemaking industry.

## 2. Results and Discussion

### 2.1. Extraction of Polyphenolic Compounds from Grape Marc

This study presents data on the extraction of some polyphenolic compounds with potential photoprotective effects from grape pomace, a by-product of the vinification process of red grapes, including varieties such as Fetească Neagră, Blaufränkisch, Merlot, and Isabella. The aim was to obtain pomace extracts that could be valorized in the cosmetic industry. The first three varieties are noble grape species grown in large areas of Romanian vineyards. Isabella is a HDP (hybrid direct producer) variety originating from the USA, known especially for its high contents of phenolic acids [64]. 

**Figure 2:**
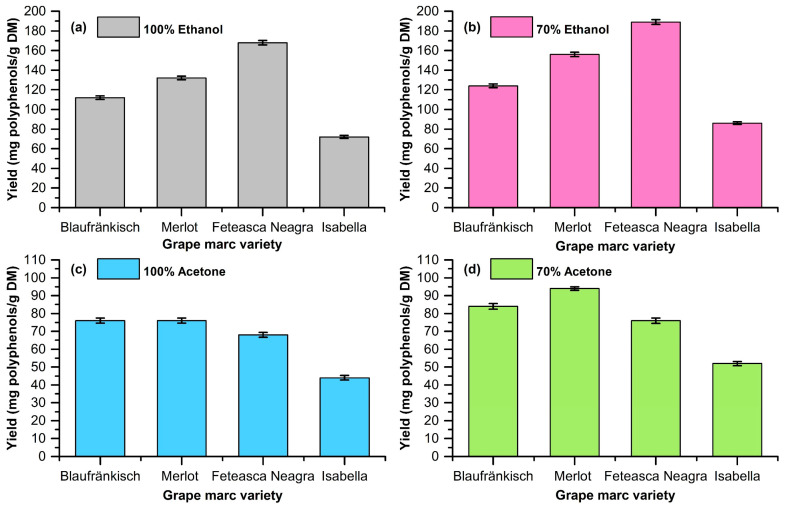
Experimental data regarding the extraction yield depending on the solvent (**a**) 100% ethanol; (**b**) 70% ethanol; (**c**) 100% acetone; (**d**) 70% acetone.

**Figure 3:**
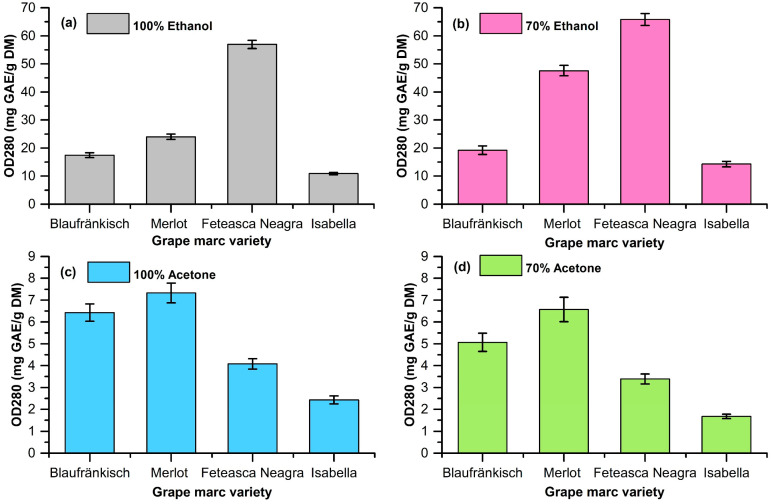
Experimental data for the OD280 index for extracts (**a**) 100% ethanol; (**b**) 70% ethanol; (**c**) 100% acetone; (**d**) 70% acetone.

**Figure 4:**
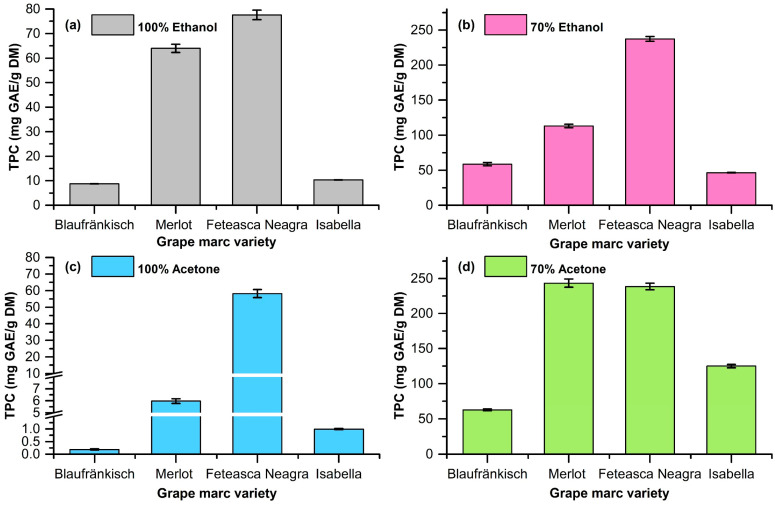
Experimental data for the total polyphenolic content (TPC) for extracts (**a**) 100% ethanol; (**b**) 70% ethanol; (**c**) 100% acetone; (**d**) 70% acetone.

The extractions were carried out using ethanol and acetone at concentrations of 70% and 100% as solvents. The plant-material-to-solvent ratio was 1/10 (*w*/*v*). The stirring rate during the extraction was 800 rpm. The extraction was performed at a temperature of 40 °C for 2 h. The mixture was then separated via centrifugation, and the supernatant was analyzed in order to determine the OD280 index, the TPC via the Folin–Ciocalteu method, and the antioxidant activity via the CUPRAC assay. The determination of the extraction yield was carried out via evaporation to dryness, under vacuum, and the remaining solid was weighed and related to the amount of initial dried grape marc (DM). The results are shown in Table 1 and Figure 2, Figure 3 and Figure 4. 

**Figure 5:**
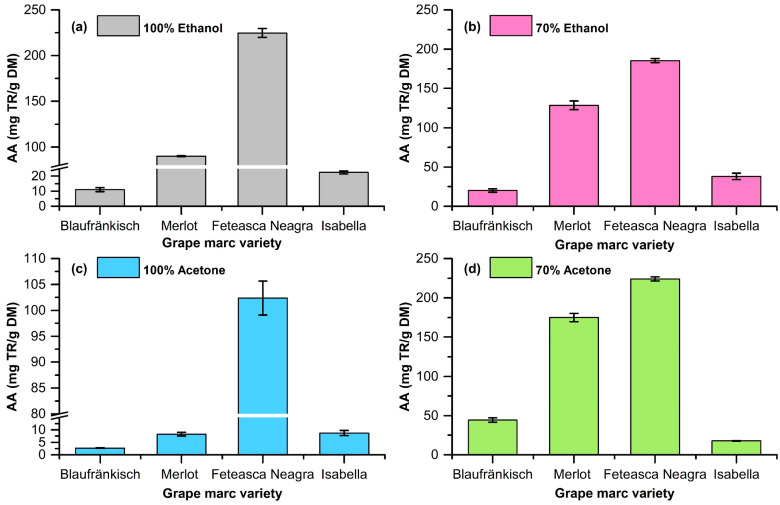
Experimental data for the antioxidant activity for extracts (**a**) 100% ethanol; (**b**) 70% ethanol; (**c**) 100% acetone; (**d**) 70% acetone.

The obtained data highlight the fact that the extraction of polyphenolic compounds from grape marc is dependent on the pomace type and the type of extraction solvent (see Figure 2, Figure 3, Figure 4 and Figure 5). 

**Figure 6:**
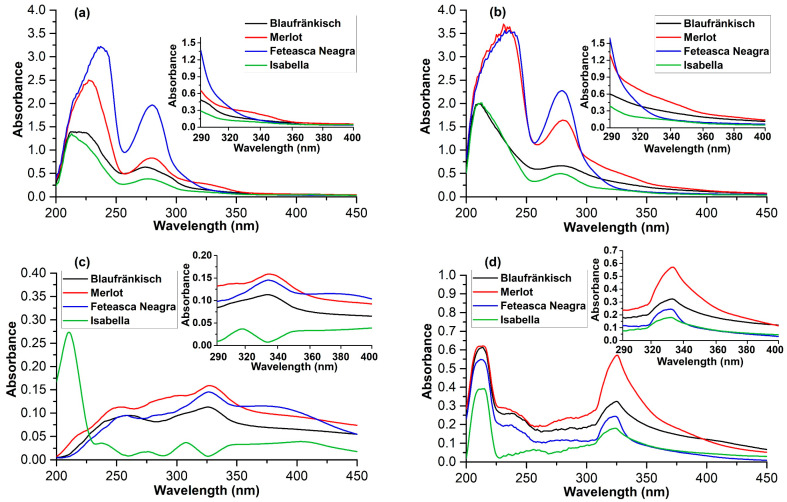
The UV-Vis spectra of the extracts (conc. 10 mg/mL) (**a**) 100% ethanol; (**b**) 70% ethanol; (**c**) 100% acetone; (**d**) 70% acetone.

The UV-VIS absorption spectra of the extracts (concentration of 10 mg/mL) in the 290–400 nm range are also presented for all extracts (Figure 6). This analysis provides an overview of the photoprotective effect against UV radiation. 

The highest extraction yields are obtained for grape marc from Fetească Neagră, followed by Merlot, Blaufränkisch, and Isabella. The solvent that ensures the best extraction in all cases is 70% ethanol. Ethanol ensures a better extraction yield compared to acetone. The addition of water has a beneficial effect for both solvents, increasing the amount of extracted polyphenolic compounds. The best extraction yield is obtained in the case of 70% ethanol extraction of Fetească Neagră grape marc (189 ± 2.34 mg/g DM).

In UV spectrophotometry, 280 nm is the analytical wavelength for the evaluation of total phenolics. However, studies have shown that not all polyphenols are detectable at this wavelength [65]. If gallic acid shows an intense band at 280 nm, those polyphenolics (cinnamic acid derivatives or chalcones, for example) have a characteristic absorption band located at 320 nm). 

Flavonols (quercetin and rutin) have an absorption band shifted towards 360 nm. Also, when glycosylated or esterified with polyphenolic acids, anthocyanins tend to shift the absorption maximum above 300 nm.

The analysis of the extracts based on the OD280 index shows that the best results in terms of extraction are obtained when using 70% ethanol, followed by 100% ethanol. The grape marc varieties that lead to a high OD280 index are Fetească Neagră (65.84 ± 2.13 mg GAE/g DM in 70% ethanol and 56.95 ± 1.45 mg GAE/g DM in 100% ethanol) and Merlot (47.59 ± 1.88 mg GAE/g DM in 70% ethanol). Extractions with 100% or 70% acetone lead to low OD280 values in all cases.

The Folin–Ciocalteu method is based on the redox properties of polyphenols. This method quantitatively analyzes all phenolic molecules without making any differentiation between gallic acid, monomers, dimers, and large phenolic compounds. The analysis carried out on the obtained extracts shows that the highest content in polyphenols is obtained in the case of the 70% acetone extraction of Fetească Neagră pomace (238.62 ± 4.83 mg GAE/g DM), followed by the 70% ethanol extraction of the same raw material. In all cases, the extraction with 70% acetone leads to high TPC values compared to the other solvents. The use of 70% aqueous acetone solution leads to us obtaining mixtures rich in polyphenols but with low absorbance in the UV range (Figure 5). In addition, a series of compounds that absorbs in the visible range is extracted. This shows that acetone favors the extraction of condensed products (tannins, etc.). Regarding the raw material, the grape marc obtained from Fetească Neagră is the one with the highest content of polyphenolic compounds, demonstrated for all varied parameters, followed by Merlot, Blaufränkisch, and Isabella variety.

The evaluation via the CUPRAC method highlights the fact that the Fetească Neagră marc has a high content of polyphenolic compounds with high antioxidant activity. Extraction with acetone leads to high values of antioxidant activity for each type of grape marc, confirming what was stated above. From the point of view of antioxidant activity, the Merlot pomace ranks second, while the values for the hybrid variety Isabella are low. Extraction with 70% ethanol also leads to extracts with high values of antioxidant activity.

Regarding the concentration of polyphenolic compounds with the highest absorbance in the UV range (290–400 nm), better results are achieved in the case of Merlot marc (Figure 6). The extraction with 70% ethanol leads to the highest absorbance value. Although the extraction with 70% acetone exhibits higher polyphenolic compound concentration values, they display lower absorbance in the UV range. As shown in Figure 6, the absorption maxima are shifted bathochromically towards the visible range. Thus, they are probably polymer structures with a lower potential photoprotective effect.

### 2.2. Spectrophotometric Analysis of the Photoprotective Effect of Grape Marc Extracts

The photoprotective effect evaluation of the previously obtained grape marc extracts was carried out based on the method developed by Mansur [66]. The extracts were obtained by following the procedure outlined by this method, which involves extraction with a 10 times larger volume of solvent. SPF_spectrophotometric_ represents the magnitude of the extract absorbance in the UVB range compared to a standard sunscreen formulation containing 8% homosalate, which presented a SPF value of 4. For in vitro spectrophotometric methods, SPF_spectrophotometric_ was initially defined as the reciprocal of transmittance of erythematous light [67]. This means that a sunscreen product that transmits all erythema light must have an SPF of 1.0, while one which absorbs all light must have an SPF of infinity. The Mansur method uses the absorbance of some solutions measured in the UVB range and is a measure of the amount of UV radiation capable of producing sunburn retained by compounds with a photoprotective effect.

The analysis was performed considering the UV-VIS spectra measured on diluted extracts with a concentration of 10 mg/mL (Figure 6). The spectra were measured in the 200–450 nm range, but only the data from the 290–320 nm interval were used in the photoprotective effect calculation. The obtained results are presented in Table 2.

**Figure 7:**
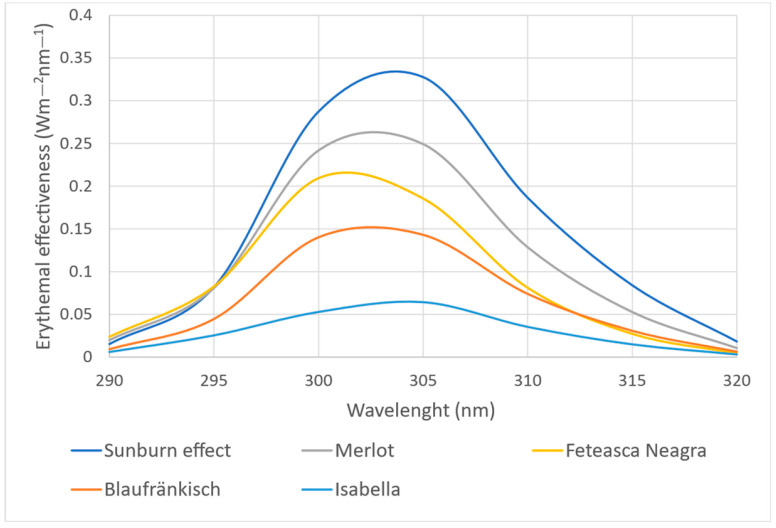
The photoprotective spectral efficiency of the grape marc extracts.

The data analysis shows that the best results, considering the presence of compounds capable of absorbing UV radiation (290–320 nm), are achieved when the extraction is carried out with 70% ethanol. In addition, the variety that contains these compounds in the highest amount is Merlot, followed by Fetească Neagră and Blaufränkisch. The sun protection factor estimated via the Mansur method for 70% ethanol extraction from the Merlot variety is 7.83 ± 0.76. Figure 7 shows the comparative spectral analysis of the extracts obtained from the four varieties of grape marc obtained using 70% ethanol regarding the photoprotective effect quantified via the Mansur method. Sunburn effect represents the irradiance in the UVB zone (E(λ)·I(λ)) necessary to produce sunburn. The protection is demonstrated by the ability of the compounds in the extract to absorb as much of this irradiance as possible. As a numerical value, it represents the area under the erythemal effectiveness curve (E(λ)·I(λ)·A(λ)) related to the area under the irradiation curve (sunburn effect) expressed as a percentage. 

It can be noticed that for the extract obtained using the Merlot grape marc, which has the highest SPF_spectrophotometric_ value, the sunburn effect is reduced by approximately 70%.

### 2.3. Obtaining Sun Protection Creams and In Vitro Analysis of Their Photoprotective Effects 

The possibility of using extracts rich in polyphenolic compounds in the preparation of sun protection creams was tested using the 70% ethanol extract obtained from the Merlot marc. Regarding the absorbance in the UV range and the SPF_spectrophotometric_ factor determined via the Mansur method, the above-mentioned extract showed the best results. 

The solution used in the preparation of creams with photoprotective effects was obtained by redissolving the solid resulting from the Merlot pomace extraction (the solid was obtained as described in Section 3) in either ethanol or water. The concentration of these solutions was 39 mg of solid/mL of solvent.

**Figure 8:**
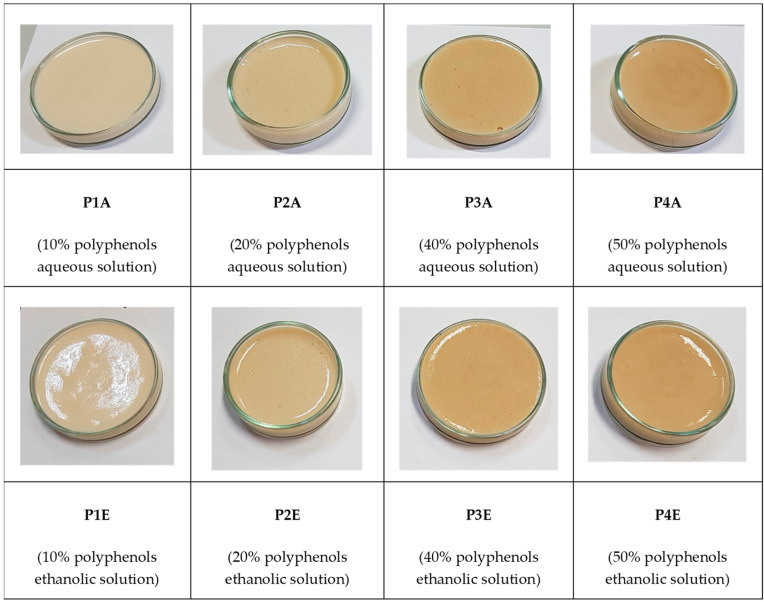
Creams with a potential photoprotective effect prepared with Merlot pomace extract.

Eight samples containing variable concentrations of polyphenolic compounds added as an aqueous or ethanolic solution were prepared. In Figure 8, the sun protection creams containing the Merlot pomace extract are shown.

The creams stability was evaluated via visual examination at 24 and 48 h. The analysis of the potential photoprotective effect was carried out via the COLIPA method using the Transpore 3M surgical adhesive tape as support. It mimics the topography of the human skin surface, being provided with pores to ensure absorption and water permeability in the same way as human skin. Table 3 shows the experimental data obtained for the prepared sunscreen lotions.

The compatibility of the polyphenolic extracts with the lotion base is very good when using aqueous solutions and good for the ethanolic ones. At this stage, the tested method for obtaining the creams allows the addition of up to 50% and 40% aqueous and ethanolic extract solutions, respectively. The stability of the emulsion is influenced by the addition of the polyphenolic solution. With aqueous solutions, there is a tendency for phase separation at a lotion/polyphenolic solution ratio of 1:1 (*w*/*w*). For ethanolic solutions of polyphenols, the tendency for phase separation is observable at a lotion/polyphenolic solution ratio of 1:0.2 (*w*/*w*), which becomes distinct at a ratio of 1:1 (*w*/*w*). The color of the lotion base is light beige. The addition of polyphenolic extracts leads to a bathochromic effect, which is more significant with higher amounts of solution being added. Compared to the initial color of the extracts, the change is not significant, and the creams maintain an acceptable visual appearance.

**Figure 9:**
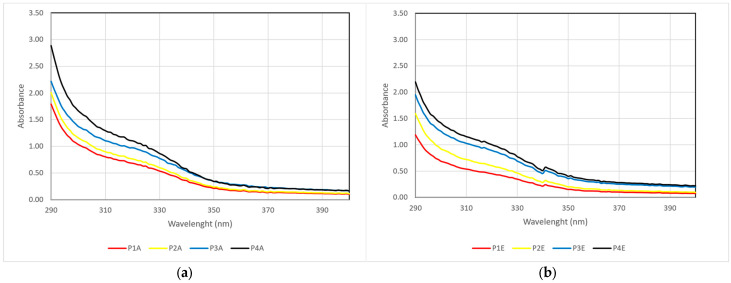
Absorption spectra of sun protection creams in the UV range (**a**) with aqueous polyphenolic extracts; (**b**) with ethanolic polyphenolic extracts.

**Figure 10:**
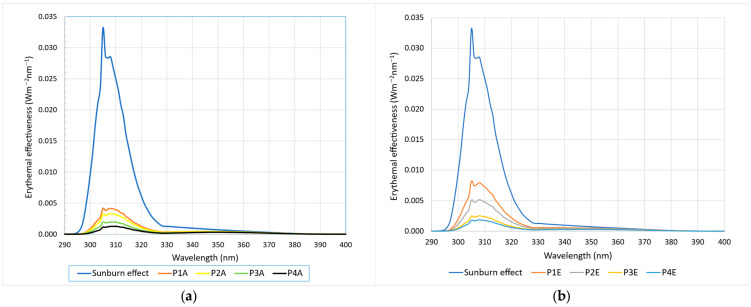
The photoprotective effects of sunscreens with polyphenolic extracts resulting from the analysis via the COLIPA method, (**a**) with aqueous polyphenolic extracts; (**b**) with ethanolic polyphenolic extracts.

The analysis of the results obtained through the in vitro determinations based on the COLIPA method shows a potential photoprotective effect for the polyphenolic compounds extracted from grape marc. Figure 9 and Figure 10 show the data regarding the spectral analysis of creams with a photoprotective effect obtained using an aqueous extract and an ethanolic one, respectively.

In both cases, the protection factor increases with the increase in the polyphenol concentration, the maximum SPF_in vitro_ value (14.07 ± 1.50) being obtained when 50% aqueous polyphenolic extract is added to the lotion base. For the ethanolic extract, the value of the in vitro SPF factor for the 50% concentration is 11.46 ± 1.32. In all cases, the creams obtained with ethanolic extracts show lower in vitro SPF values than the creams obtained with aqueous extracts at the same concentrations.

UVAPF_0_ values are slightly higher in the case of ethanolic extracts compared with aqueous extracts, which shows a higher protection for the UVA range, while the aqueous extracts protect better in the UVB area. A possible cause is the bathochromic shift of the absorption due to a solvatochromic effect. 

Figure 10 shows the erythemal effectiveness of the prepared sun creams. It is observed that there is a significant reduction in the impact of UV radiation, reducing the risk of sunburn. For example, for the cream with the highest SPF value (14.07 ± 1.50), the absorption of UV radiation is 92.89% (see Supplementary Materials).

### 2.4. Study Limitations and Development Directions

The recommendations of the European Commission regarding the effectiveness of sun creams show that a product with an SPF (determined via in vitro methods) of over 15 provides average protection. Additionally, the UVAPF0 value must be at least 1/3 of the SPF value [68]. Dermatologists use several criteria to recommend sunscreen, including the SPF level (99%), broad-spectrum protection (96%), cosmetic perception (71%), and photostability (42%) [69]. An SPF 15 sunscreen absorbs 93.3% of radiation that induces erythema, an SPF 30 sunscreen absorbs 96.7%, and an SPF 50 sunscreen absorbs about 98% of UVB radiation [68].

Compared to these requirements, the SPF values estimated via in vitro methods are quite low. One possibility when using polyphenolic extracts to obtain sun protection creams is to combine them with synthetic UV absorbers (which will be the subject of future studies). This will lead to the reduction in their concentration and, implicitly, the side effects that they produce. At the same time, the addition of natural polyphenolic compounds with an antioxidant effect can bring supplementary benefits by eliminating free radicals and inhibiting enzymes. The latter impacts the anti-inflammatory pathways, leading to less erythema. 

The extraction of polyphenolic compounds from grape marc for use in the production of cosmetic products with a photoprotective effect represents a viable alternative for the valorization of this waste. They are available in large quantities, and a new way of valorization is welcome, bringing added value for producers in the wine and winemaking industry.

Also, the study carried out is an estimate based especially on spectral analysis. The real data regarding the solar protection of such products can only be obtained after performing an in vivo study.

## 3. Materials and Methods

### 3.1. Materials

The grape marc (2023 production) used for the extraction of polyphenols is the by-product resulting from the vinification of black grapes of different varieties: Fetească Neagră, Blaufränkisch, Merlot, and Isabella. The grape marc of the latter was provided by a private farm from Dambovita area. The others were supplied from the Dealu Mare vineyard of Research Institute for Viticulture and Oenology Valea Calugareasca, Romania.

The winemaking process begins with must production, which consists of harvesting, destemming, crushing, and pressing the grapes. Subsequently, the must enters the fermentation process. Grape marc (5 kg from each variety) was acquired from the producer immediately after must production. The pomace was refrigerated for 24 h before being subjected to drying. The grape marc was dried in an oven at 50 °C for 24–36 h, then ground using an electric grinder. The resulting powders were stored in polyethylene bags in a cool, dry, and dark place.

The chemicals used in this study were ethanol (99.50%, p.a, Chimreactiv SRL, Bucharest, Romania), acetone (99.8%, AnalaR NORMAPUR) and the Folin–Ciocalteu reagent (VWR Chemicals, Wien, Austria), gallic acid (98%), neocuproine (98%), copper (II) chloride (99), ammonium acetate (97%), and 6-hydroxy-2,5,7,8-tetramethylchroman-2-carboxylic acid (TROLOX) (97%, Sigma-Aldrich, Schnelldorf, Germany).

The commercial lotion base (Lotion Base Organic) used, an O/W emulsion, is produced by the company Elemental SRL Oradea, Romania and contains *Aloe barbadensis* leaf juice, water, *Cocos nucifera* oil, *Helianthus annuus* seeds, cetylaryl alcohol, coco-glucoside, *Butyrospermum parkii* butter, potassium sorbate, xanthan gum, *Prunus armeniaca* kernel oil, sodium benzoate, citric acid, tocopherol, and lactic acid. Transpore 3M (3M HealthCare, Calarasi, Romania) surgical adhesive tape was also utilized.

### 3.2. Extraction of Polyphenols from Grape Marc

The extraction was carried out in a 100 mL round-bottomed glass flask connected to an ascending condenser and a Pt100 temperature probe on a CAT KM16.4D magnetic stirring and heating device (CAT, Ballrechten-Dottingen, Germany). The volume of solvent used for extraction was 50 mL. The extraction solvents were ethanol and acetone at concentrations of either 100% or 70% in water. The plant material to solvent ratio was 1/10 (*w*/*v*). The stirring rate during the extraction was 800 rpm. The extraction was performed at a temperature of 40 °C for 2 h. The mixture was then separated via centrifugation, and the supernatant was analyzed in order to determine the total phenolic content and the antioxidant activity. To determine the extraction yield, the extract was evaporated to dryness, under vacuum, and the remaining solid was weighed and related to the amount of initial dry grape marc (5 g).

### 3.3. Determination of Total Polyphenolics OD280 Index 

The quantification of total polyphenols was performed spectrophotometrically via OD280 index assessment. The method is based on the property of benzene rings, characteristic of phenolic compounds, to absorb in the ultraviolet range, registering a maximum at 280 nm. The grape marc extract was diluted in a ratio of 1 to 100. Further, the absorbance of the diluted extract was measured at a wavelength of 280 nm. The measurements were carried out using a Jasco V550 UV-VIS spectrophotometer (ABL&E-JASCO, Cluj-Napoca, Romania), compared to distilled water. The OD280 index of total polyphenols was quantified as milligrams of gallic acid equivalents per 1 g of dry matter (mg GAE/g DM), using a standard curve corresponding to 0.019–0.1 mg/mL gallic acid solution [70]. 

### 3.4. Determination of Total Phenolic Content 

A second quantification of the total polyphenolic content was carried out using the Folin–Ciocalteu method [71,72]. The analysis of the polyphenolic extracts was carried out by adding 1 mL of the Folin–Ciocalteu reagent, 3 mL of distilled water, a 0.1–0.5 mL sample (depending on the sample concentration), and 1.5 mL of 20% Na_2_CO_3_ solution to a 10 mL volumetric flask. Further, the mixture was diluted to 10 mL with distilled water. The samples were kept in the dark for 30 min. The absorbance was measured at a wavelength of 765 nm using a Jasco V550 UV-VIS spectrophotometer, compared to distilled water. The results were quantified as milligrams of gallic acid equivalents per 1 g of dry matter (mg GAE/g DM) using a standard curve corresponding to 0–1.26 mg/mL gallic acid solution. 

### 3.5. Determination of Antioxidant Activity 

The analysis of the antioxidant activity was carried out by CUPRAC assay (CUPric Reducing Antioxidant Capacity) [73,74], which is based on the reduction of Cu (II) ions due to the antioxidant activity of phenolic compounds. To determine the antioxidant activity, a 10^−2^ M CuCl_2_ solution, a 1.0 M ammonium acetate (NH_4_Ac) (pH ± 7.0) buffer solution, and a 7.5 × 10^−3^ M neocuproine (Nc) solution were prepared.

To analyze the sample, 1 mL each of the CuCl_2_, Nc, and NH_4_Ac solutions; x mL of sample; and (1.1-x) mL of distilled water were added to a vial. The absorbance was measured after 30 min, at a wavelength of 450 nm and compared to the control (obtained from mixing the reagents without the sample). The measurements were conducted with the help of a Jasco V550 UV-VIS spectrophotometer. The antioxidant activity was quantified as milligrams of TROLOX (6-hydroxy-2,5,7,8-tetramethylchroman-2-carboxylic acid) equivalents per 1 g of dry matter (mg TE/g DM) using a standard curve corresponding to 0–0.25 mg/mL TROLOX solution. 

### 3.6. Spectrophotometric Analysis of the Photoprotective Effect of Grape Marc Extracts

The spectrophotometric analysis of the extracts is based on the research of Mansur et al. [66], who developed a very simple mathematical equation that replaces the in vitro method proposed by Sayre et al. [67]. It uses UV spectrophotometry and the following equation:

(1) $${SPF}_{spectrophotometric}=CF\cdot \sum_{290nm}^{320nm}E(\lambda )\cdot I(\lambda )\cdot A(\lambda )$$

where E(*λ*)—erythemal effect spectrum; I(*λ*)—solar intensity spectrum; A(*λ*)—the absorbance of the sunscreen product; CF—correction factor (=10). It was determined that a standard sunscreen formulation containing 8% homosalate presented a SPF value of 4 [66].

The values of E × I are constant. They were determined by Sayre [70] and are shown in Table 4.

The analysis was carried out based on the UV-VIS spectra measured every 5 nm, in the 200–450 nm range, for the solutions diluted at a concentration of 10 mg/mL. The measurements were performed in triplicate. For the analysis of the photoprotective effect, the data from the 290–320 nm range were used in the calculation.

### 3.7. Obtaining Lotions with a Photoprotective Effect Based on Grape Marc Extracts

To obtain products with a photoprotective effect, the lotion base was heated to 30–35 °C under constant stirring. Subsequently, the extract solution was added and stirred for 1 h at a temperature between 30 and 35 °C and allowed to mature at room temperature for 24 h.

### 3.8. Analysis of the Photoprotective Effect of Polyphenol Extracts Containing Lotions

The prepared creams were evaluated via visual examination at 24 and 48 h in order to determine their stability. The photoprotective effect was also evaluated via the COLIPA method [62,63]. Transpore 3M surgical adhesive tape was used as a substrate according to the version proposed by Diffey [75,76]. The tapes were mounted in 35 mm plastic photographic slide frames.

The sunscreens were applied, using a latex-gloved finger, to the non-adhesive side of the strips at an application rate of 2 mg/cm^2^ (50 ± 0.5 mg per slide). The evenness of the layer was ensured by applying the cream for 30 s in a circular motion while maintaining the upper part of the frame tightly fixed.

The plates were weighed both before and after application. The results were corrected for a quantity of 0.050 g of cream applied to the plate using the following equation:

(2) $$A_{corrected}=\frac{0.05}{m_{sample}}\times A_{measured}$$

Before measuring, the samples were allowed to rest for 30 min at room temperature in the dark. The measurements regarding the efficiency of the photoprotective effect were carried out using a Jasco V550 UV-VIS spectrophotometer with an integrating sphere. The device was calibrated via the Spectralon standard. The transmittance of the samples was measured in the 200–500 nm range. Further, the transmittance was converted into absorbance with the help of Spectra Manager II software, version 1.54.03 (build 1). The calculations regarding the sun protection factor were performed using Equation (2) for the absorbance correction and Equations (3) and (4) for SPF_in vitro_ and UVAPF_0_ calculations, respectively. The samples were measured in five different areas, and the transmittance values obtained were averaged.

(3) $${SPF}_{invitro}=\frac{\int_{\lambda =290nm}^{\lambda =400nm}E(\lambda )\cdot I(\lambda )\cdot d\lambda }{\int_{\lambda =290nm}^{\lambda =400nm}E(\lambda )\cdot I(\lambda )\cdot 10^{{-A}_{0}(\lambda )}\cdot d\lambda }$$

(4) $${UVAPF}_{0}=\frac{\int_{\lambda =320nm}^{\lambda =400nm}P(\lambda )\cdot I(\lambda )\cdot d\lambda }{\int_{\lambda =320nm}^{\lambda =400nm}P(\lambda )\cdot I(\lambda )\cdot 10^{{-A}_{0}(\lambda )}\cdot d\lambda }$$

where E(*λ*)—the erythemal action spectrum (CIE-1987); I(*λ*)—the spectral irradiance of the UV source; P(*λ*)—the PPD (Persistent Pigment Darkening) action spectrum; A_0_(*λ*)—the average monochromatic absorption measurements on the substrate of the tested product before UV exposure; d*λ*—the wavelength step (1 nm) [63].

## 4. Conclusions

The aim of this study was to establish the suitable grape marc extract required to enhance the photoprotective effect of sun protection creams. To achieve this goal, pomace of different grape varieties, namely Merlot, Fetească Neagră, Blaufränkisch, and Isabella, was used for the extraction of bioactive compounds that absorb in the UV range. Different extraction parameters, such as type of solvent (ethanol and acetone) and solvent concentration in water (100 and 70% ethanol or acetone), were studied. The resulted extracts were analyzed in order to determine different indices, namely the OD280 index, TPC, antioxidant activity, and absorbance in the UV range. Regarding the extraction efficiency, the best results were achieved when 100% and 70% ethanol was used as solvent for all studied indexes. The use of 70% aqueous acetone solution led to mixtures rich in polyphenols but with low absorbance in the UV range. The grape marc resulting from Fetească Neagră was the one with the highest content of polyphenolic compounds, as demonstrated in all cases, followed by the Merlot, Blaufränkisch, and Isabella varieties. The pomace extracts were also analyzed in order to determine the photoprotective effect via the Mansur method. This analysis showed that the highest concentration of compounds capable of absorbing UV radiation (290–320 nm) is achieved for the extract obtained from Merlot pomace with 70% ethanol as solvent. The sun protection factor estimated via the Mansur method was 7.83 ± 0.76. Thus, this extract was chosen as the most suitable one to be incorporated into the sun protection cream. The latter was analyzed to determine the stability via visual evaluation and the photoprotective effect via the COLIPA method. The analysis of the results showed a potential photoprotective effect of the cream mixed with the polyphenolic compounds extracted from grape marc. The compatibility of the polyphenolic extracts with the lotion base used in this stage was very good when an aqueous solution was used and good for the extracts in an ethanolic solution. The method of obtaining the sunscreen allowed the addition of up to 50% aqueous extract solution and up to 40% ethanolic one, indicating an in vitro-estimated sun protection factor of about 10–15. Future research directions include the optimization of the extraction process to facilitate scalability and industrial application. Additionally, a study on the properties of cosmetic products, such as stability over time, photostability, water resistance, the determination of SPF in vivo, toxicity, etc., should be conducted. Furthermore, exploring the potential of combining natural products with established synthetic organic or inorganic filters is essential to enhance the protective effect.

## Figures and Tables

**Table 1:** Experimental data for extraction of polyphenolic compounds from grape marc.

Grape Marc	Solvent	Yield (mg Polyphenols/g DM)	OD280 (mg GA/g DM)	TPC (mg GA/g DM)	Antioxidant Activity via the CUPRAC Assay (mg TROLOX/g DM)
Blaufränkisch	100% ethanol	112 ± 1.85	24.01 ± 0.94	63.99 ± 1.68	89.92 ± 0.54
	70% ethanol	124 ± 1.96	19.20 ± 1.54	58.57 ± 2.32	20.11 ± 2.22
	100% acetone	76 ± 1.43	6.43 ± 0.40	0.19 ± 0.02	2.69 ± 0.13
	70% acetone	84 ± 1.58	5.07 ± 0.42	62.54 ± 1.20	44.39 ± 2.78
Merlot	100% ethanol	132 ± 1.85	24.01 ± 0.94	63.99 ± 1.68	89.92 ± 0.54
	70% ethanol	156 ± 2.20	47.59 ± 1.88	113.12 ± 2.39	128.50 ± 5.55
	100% acetone	76 ± 1.44	7.33 ± 0.45	5.98 ± 0.19	8.26 ± 0.76
	70% acetone	94 ± 1.02	6.58 ± 0.56	243.39 ± 5.88	174.85 ± 5.24
Fetească Neagră	100% ethanol	168 ± 2.26	56.95 ± 1.45	77.60 ± 1.92	224.77 ± 4.94
	70% ethanol	189 ± 2.34	65.84 ± 2.13	237.35 ± 3.44	185.40 ± 2.43
	100% acetone	68 ± 1.34	4.08 ± 0.24	58.20 ± 2.39	102.36 ± 3.28
	70% acetone	76 ± 1.45	3.39 ± 0.23	238.62 ± 4.83	223.98 ± 2.52
Isabella	100% ethanol	72 ± 1.65	10.89 ± 0.40	10.37 ± 0.05	22.52 ± 0.96
	70% ethanol	86 ± 1.46	14.28 ± 0.98	46.48 ± 0.34	38.01 ± 4.13
	100% acetone	44 ± 1.34	2.43 ± 0.18	0.99 ± 0.02	8.69 ± 1.03
	70% acetone	52 ± 1.16	1.67 ± 0.10	125.08 ± 2.39	17.83 ± 0.30

**Table 2:** Data on the photoprotective effect of extracts using the Mansur method.

Variety	SPF_spectrophotometric_			
	100% Ethanol	100% Acetone	70% Ethanol	70% Acetone
Blaufränkisch	2.76 ± 0.24	0.97 ± 0.12	4.33 ± 0.46	2.05 ± 0.16
Merlot	3.89 ± 0.42	1.38 ± 0.23	7.83 ± 0.76	2.89 ± 0.22
Fetească Neagră	5.19 ± 0.52	1.12 ± 0.32	6.15 ± 0.58	1.30 ± 0.23
Isabella	1.60 ± 0.60	0.29 ± 0.08	2.24 ± 0.32	1.03 ± 0.15

**Table 3:** Experimental results of obtained sun protection creams performances.

Sample	Stability—24 Ore	Stability—48 Ore	SPF In Vitro	UVAPF0
P1A	Very good	Very good	5.65 ± 0.87	1.63 ± 0.35
P2A	Very good	Very good	6.84 ± 0.92	1.72 ± 0.24
P3A	Very good	Very good	10.46 ± 1.10	2.15 ± 0.44
P4A	Good	Good	14.07 ± 1.50	2.15 ± 0.38
P1E	Very good	Very good	3.27 ± 0.87	1.42 ± 0.32
P2E	Good	Good	4.77 ± 0.94	1.59 ± 0.34
P3E	Good	Good	8.94 ± 1.10	2.21 ± 0.43
P4E	Good	Poor	11.46 ± 1.32	2.42 ± 0.46

**Table 4:** The normalized product function used in the SPF calculation.

Wavelength (nm)	E × I
290	0.0150
295	0.0817
300	0.2874
305	0.3278
310	0.1862
315	0.0839
320	0.0180
Total	1

## Data Availability

All of the data are contained within the article.

## Supplementary Materials

The following supporting information can be downloaded via this link: https://www.mdpi.com/article/10.3390/molecules29122775/s1, SPF in vitro calculation.
